# A case report of Indium lung with progressive emphysema and fibrosis underwent lung unilateral transplantation 20 years after the end of the exposure

**DOI:** 10.1186/s13000-023-01303-1

**Published:** 2023-01-28

**Authors:** Chihiro Inoue, Shinya Ohkouchi, Tatsuya Chonan, Atsuko Amata, Takashi Hirama, Ryoko Saito-Koyama, Yoshinori Kawabata, Takashi Suzuki, Yoshinori Okada, Akiyo Tanaka, Hajime Kurosawa

**Affiliations:** 1 Department of Anatomic Pathology, Graduate School of Medicine, Tohoku University, Sendai, Japan; 2 Department of Occupational Health, Graduate School of Medicine, Tohoku University, Sendai, Japan; 3 Department of Medicine, Nikko Memorial Hospital, Hitachi, Japan; 4 Department of Thoracic Surgery, Institute of Development, Aging and Cancer, Tohoku University, Sendai, Japan; 5 Department of Pathology, National Hospital Organization, Sendai Medical Center, Sendai, Japan; 6 Division of Diagnostic Pathology, Saitama Prefectural Cardiovascular and Respiratory Center, Kumagaya, Japan; 7 Environmental Medicine, Graduate School of Medical Sciences, Kyushu University, Fukuoka, Japan

**Keywords:** Case report, Indium lung, Transplantation, Emphysema, Fibrosis

## Abstract

**Background:**

Indium lung is characterized by interstitial pneumonia and/or emphysema which occurs in indium-tin oxide (ITO) workers. Indium lung is now known to progress after stopping exposure to ITO, but the long-term influences of ITO remain unclear.

**Case presentation:**

Forty seven years old, a never-smoker, who had been engaged in an ITO manufacturing process for 8 years. Emphysema was indicated by the medical check-up for ex-ITO workers, and he was diagnosed with indium lung. He underwent partial lung resections for pneumothorax two times, and obstructive pulmonary dysfunction had progressed through the years. He underwent right single lung transplant 20 years after ITO exposure. Pathologically, his lung showed severe distal acinar emphysema and honeycomb change. Fibrosis and destruction of the lung tissue significantly progressed compared to the previous partial resections. Scanning electron microscopy combined with energy dispersive spectroscopy revealed that the deposited particles contained indium and tin. After the transplantation, his respiratory function was improved.

**Conclusions:**

In this case, ITO resided in the lung tissue for 20 years, and lung tissue destruction kept progressing. Careful medical follow-up is recommended for ITO-workers even if they are asymptomatic.

## Background

Indium-tin oxide (ITO) is used as the transparent electrodes in flat-panel displays.

The first reported case of indium lung was a 27-year-old man who had worked surface grinding of the ITO targets between the ages of 23 and 26 and died of interstitial pneumonia [1]. The professional committee which supervised the occupational hygiene of the factory suggested the possibility that the inhaled ITO dust could have caused lung damage, including interstitial pneumonia and emphysema [2, 3]. Indium lung develops months to years after exposure to ITO dust and could cause devastating lung disease and respiratory failure. Indium lung is characterized by interstitial pneumonia and/or emphysema in the early stages; interstitial changes become less prominent and emphysematous changes become more prominent on radiological imaging in the disease progress [4, 5]. In pulmonary function tests, the decrease in forced expiratory volume in one second / forced vital capacity (FEV1/FVC) and the impaired lung diffusing capacity for carbon monoxide (DLco) are observed in severe cases. Characteristic pathological features are emphysema and pulmonary fibrosis with cholesterol crystal-containing granulomas and the presence of fine particles in alveolar macrophages and giant cells [5], but the mechanisms for the development of these features are still unclear. There has been one report which described the histopathology of the recipient’s lungs resected bilaterally from an ex-smoking indium lung patient [6]. We herein explored the histopathology of a non-smoker recipient indium lung resected from the patient who underwent right single lung transplantation (LTX) for indium lung, which progressed for about 20 years after stopping the exposure.

This patient had undergone partial resection of both lungs, separately at different time points, respectively, to treat refractory pneumothoraxes. The comparisons between the two sets of specimens should give us the important information on the chronological changes of the indium lung, especially on the pathogenesis of emphysema in indium lung.

## Case presentation

**Fig. 1:**
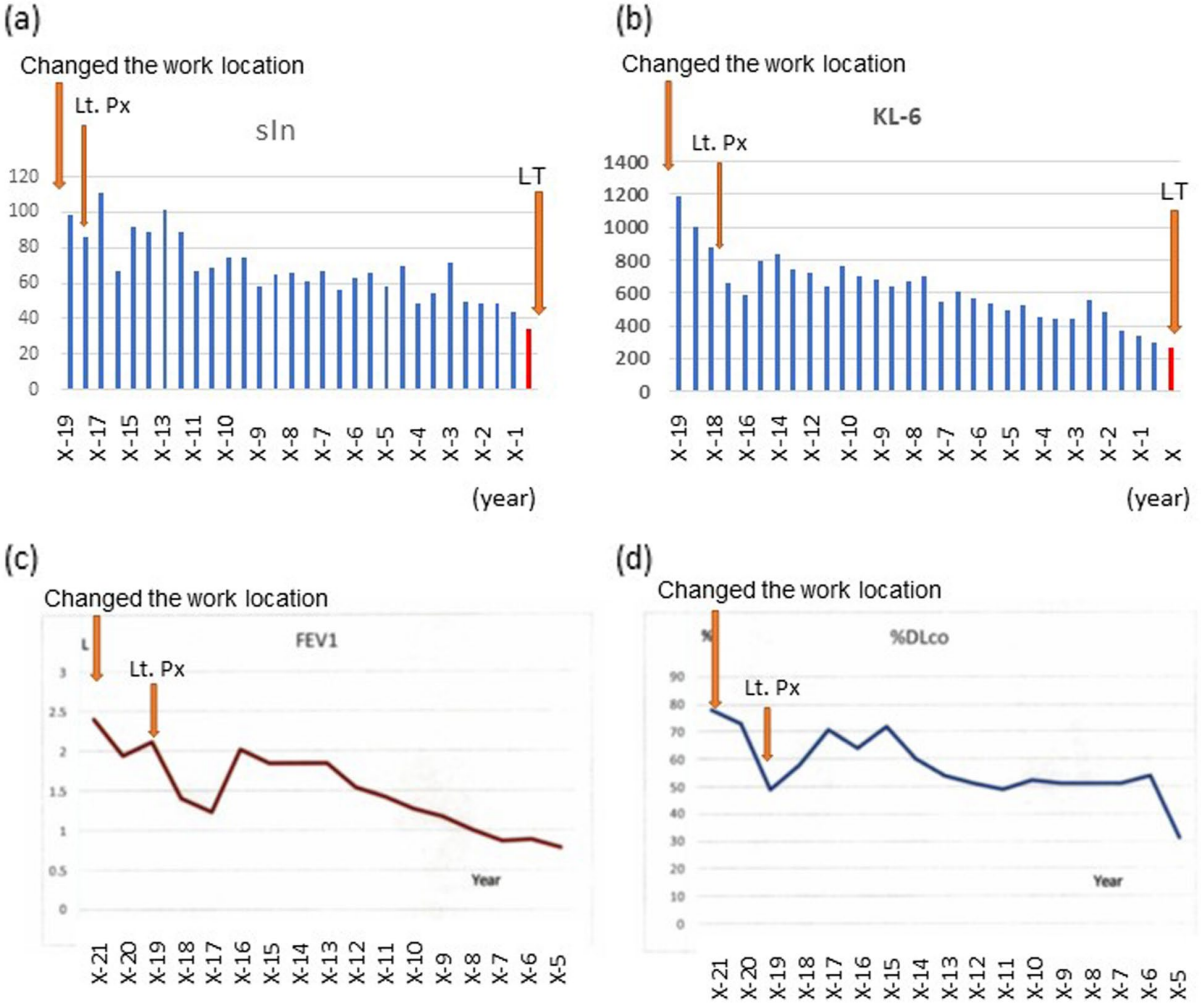
Chronological changes of serum indium, Krebs von den lungen 6, and respiratory functions. Chronological changes in (**a**) serum indium (sIn), **b** Krebs von den lungen 6 (KL-6), **c** Forced Expiratory Volume in one second (FEV1), and (**d**) lung diffusing capacity for carbon monoxide (DLco). Lt. Px.: left pneumothorax. LT: lung transplantation

**Fig. 2:**
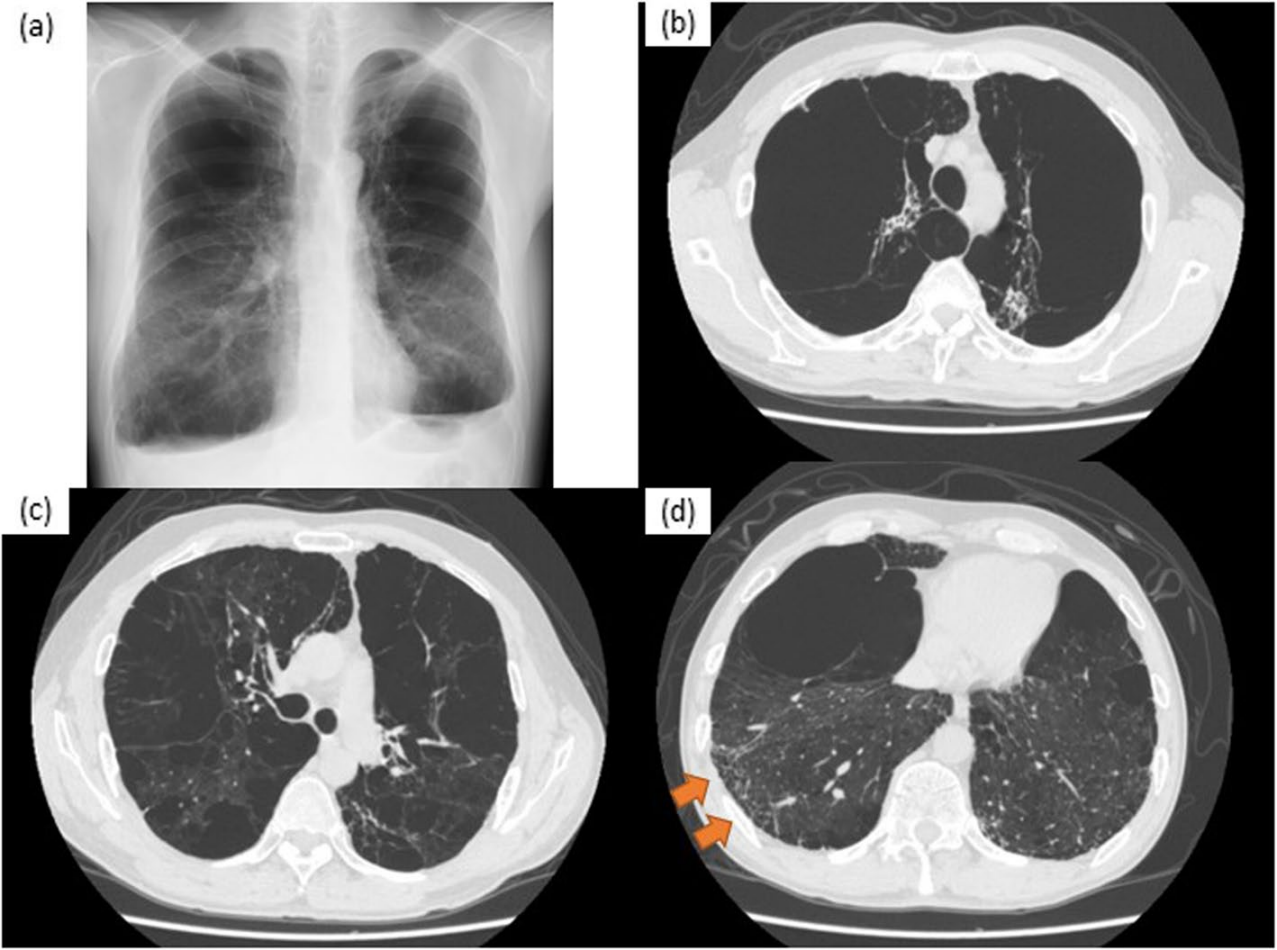
Images of chest X-ray and CT begore the lung transplantation. Images of chest X-ray (a) and CT (b)-(d). **a** The chest X-ray showed hyperinflation of bilateral lungs and a flattened diaphragm. **b**-**d** Cystic enlargement of airspace was more severe in the upper lobes than in the lower lobes. Reticular changes (orange arrows) were observed in the subpleural area in the area of ground-glass opacity mainly in the lower lobes

A 47-year-old man with no history of smoking underwent right single LTX. He was engaged in an ITO manufacturing process from the age of 18 to 26. He underwent video-assisted thoracoscopic surgery (VATS) for right apical pneumothorax in X-21(year), since then he was transferred to non-indium desk work. One of his colleagues died of severe interstitial pneumonia in X-20. Following this incident, a cross-sectional study was conducted for all the employees involved in the ITO processing. Although VC was maintained (VC of 4.89 L), FEV1 was decreased (FEV1 of 2.40 L). Serum indium concentration and Krebs von den lungen 6 (KL-6) were significantly elevated (serum indium: 99 ng/mL, normal value 0.06 ± 0.03 ng/mL; KL-6: 1,190 U/mL, normal value < 500 U/mL). High-resolution computerized tomography (HRCT) showed reticulo-nodular shadow, ground glass opacity (GGO), and mild to moderate low attenuation areas on HRCT, which suggest emphysema. Lung disease due to ITO was suspected based on the features described above. He underwent the second VATS for left pneumothorax in X-17. Although serum indium and KL-6 levels declined slowly (Fig. 1a, b), obstructive pulmonary dysfunction progressed through the years (Fig. 1c, d). After the informed consent, he was listed for LTX at Tohoku University Hospital in X-2, and underwent right single LTX in X. The LTX was uneventful. His respiratory function one year before the LTX was FVC of 4.17 L (89% of predicted), FEV1 of 0.62 L (15% of predicted) and an FEV1-to-FVC ratio of 19%. Chest radiograph and HRCT showed multiple cystic changes, severe emphysema, and reticular-nodular shadow (Fig. 2a-d). After the LTX, His lung function improved significantly to VC of 2.98 L (65% of predicted) and FEV1 of 2.17 L (55% predicted), and oxygen therapy was discontinued. Anti-granulocyte–macrophage colony-stimulating factor (GM-CSF) autoantibody was negative, and α1 antitrypsin was at a normal level throughout the entire course of the disease.

The pathological finding of the specimen obtained by VATS for the right pneumothorax has been reported previously [5]. It showed bullae and distal acinar emphysema. Cholesterol granulomas are observed in the peripheral lung. The surface of the cholesterol clefts was immune-positive for surfactant protein-A (SP-A), but little surfactants resided in alveoli. Mild infiltration of lymphocytes and brownish particles were observed in the stroma of bronchioles, but no fibrosis or obstructions were detected.

The specimen obtained by the second VATS for the left pneumothorax showed more severe air space enlargement than that in the first VATS specimen. The density of cholesterol cleft in cholesterol granulomas and the number of alveolar macrophages containing particles increased. Focal fibrosis with the destruction of elastic fiber framework were newly appeared.

**Fig. 3:**
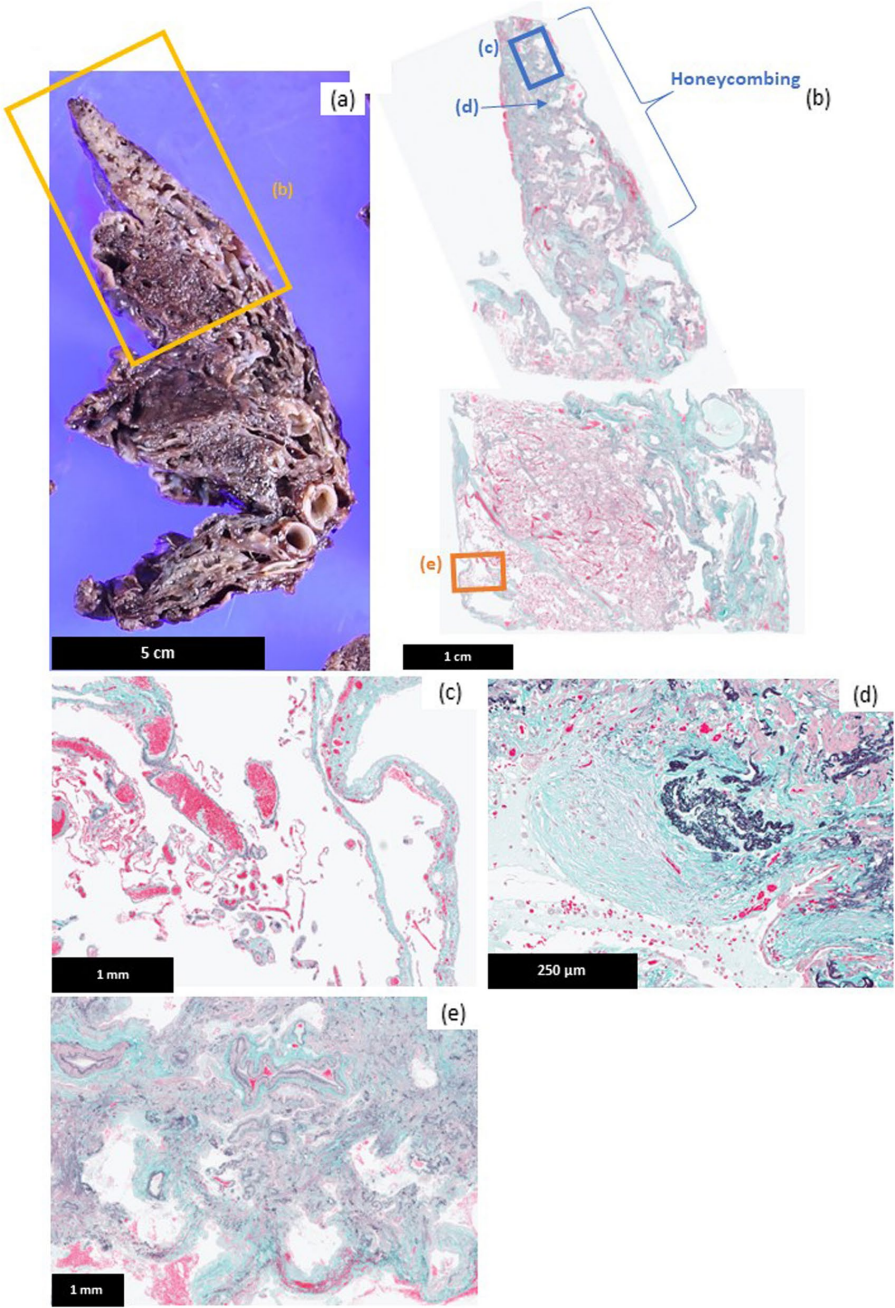
Pathological findings of the right lung resected or the lung transplantation (**a**) A macroscopic image of the upper lobe of the resected right lung. Cystic airspace enlargement and white fibrosis were observed in the subpleural area. (b)-(e) Microscopic images of the lung (Elastica-Masson stain). **b** A loupe image of an area showed a mixture of distal acinar emphysema and perilobular fibrosis and honeycombing like a usual interstitial pneumonia pattern. **c** The area with thin-walled cystic airspace enlargement showed emphysema. **d** fibroblastic foci were observed in fibrotic areas. **e** The area with severe fibrosis showed honeycomb-like change with severe destruction of lung structure

**Fig. 4:**
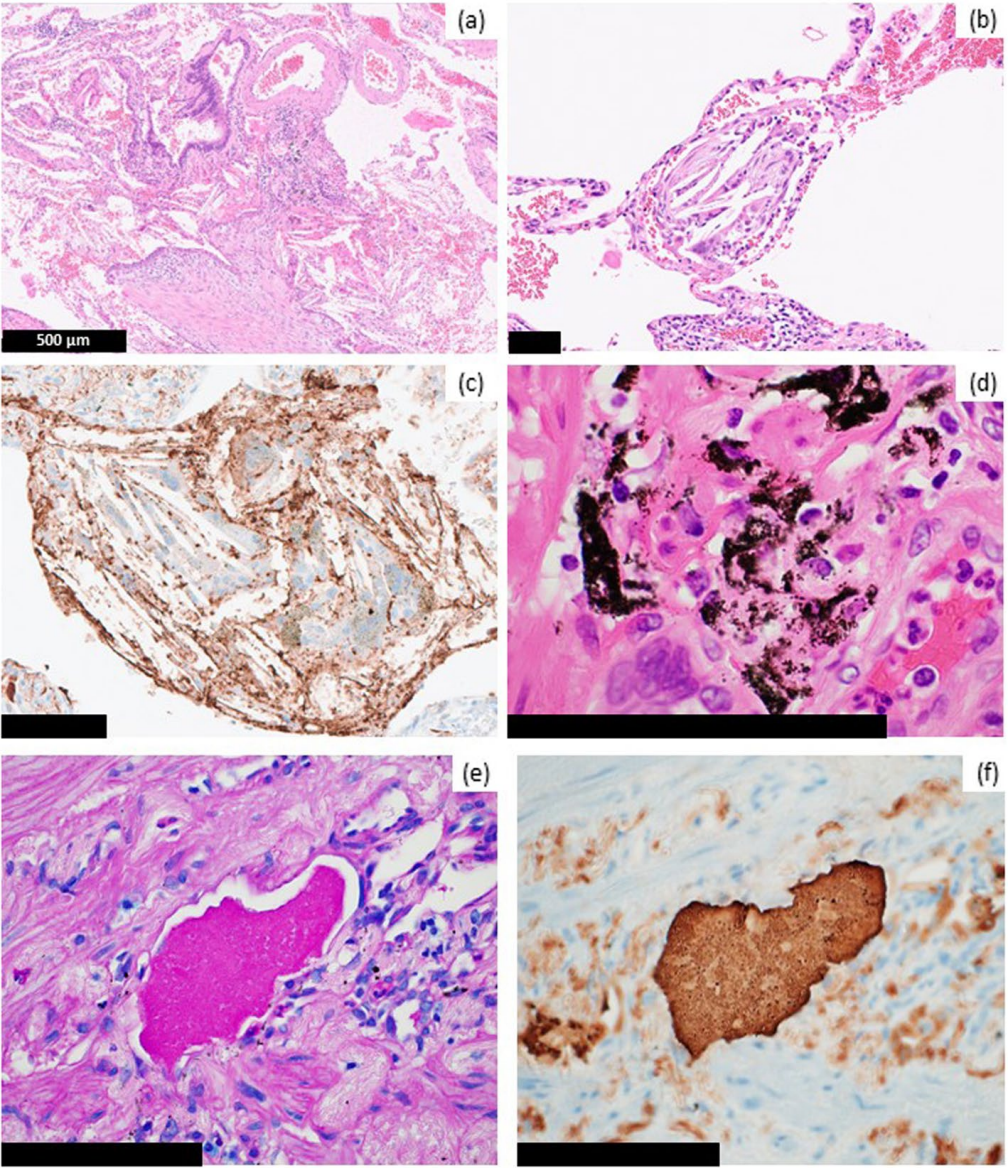
Characteristic pathological findings of indium lung observed in this case. **a** Cholesterol granulomas, which consist of cholesterol clefts, macrophages and giant cells, were observed in the air space. **b** In some cholesterol granulomas, fibrosis surrounding the granuloma was observed. **c** The surface of cholesterol clefts was immunopositive for surfactant protein-A (SP-A). **d** Black and brown particles contained clear crystal-like particles deposited in the fibrotic stroma or engulfed by macrophages. Surfactant positive for (**e**) periodic acid-Schiff (PAS) stain and (**f**) SP-A resided in the entrapped air space. (b)-(f) Bar = 100 μm

In the right lung obtained by LTX, macroscopically, prominent cystic enlargement of the airspace was observed under the pleura from the apex to the base of the lung, especially in the upper lobe. White, fibrotic changes were occasionally intermingled with the cystic airspace enlargement (Fig. 3a). Microscopically, severe distal acinar emphysema was predominantly observed in the lung (Fig. 3b, c). Residual stroma showed various degrees of fibrosis and/or elastosis. Fibroblastic foci were observed occasionally (Fig. 3d). Some fibrotic areas showed severe perilobular fibrosis and honeycomb changes similar to usual interstitial pneumonia (UIP) pattern (Fig. 3b, e). In this area, smooth muscle hyperplasia and hypertrophy were prominent (Fig. 3e). Mild to moderate inflammation was observed in alveolar septa and respiratory to terminal bronchioles (Fig. 4a). Numerous cholesterol granulomas were detected in the air space (Fig. 4a). Some of the cholesterol granulomas had fibrosis surrounding them. (Fig. 4b). The surface of cholesterol clefts was immune-positive for SP-A (Fig. 4c), as well as in those of VATs specimens. Black to brown particles was deposited in the stroma and alveolar macrophages and giant cells engulfed them in the alveoli and stroma (Fig. 4d). Some particles contained micro-sized, clear, crystal-like materials (Fig. 4d). Few particles were observed in the residual area with few pathological findings. A very small amount of surfactant, which was positive for periodic acid Schiff (PAS) stain (Fig. 4e) and immune-positive for SP-A (Fig. 4f), was detected in entrapped air space in the fibrotic area. Particles were also deposited in the hilar lymph nodes.

In summary, the histological findings of this case showed a mixture of distal acinar emphysema, severe perilobular fibrosis showing honeycomb changes. Deposition of particles and numerous cholesterol granulomas supported the diagnosis of indium lung.

**Fig. 5:**
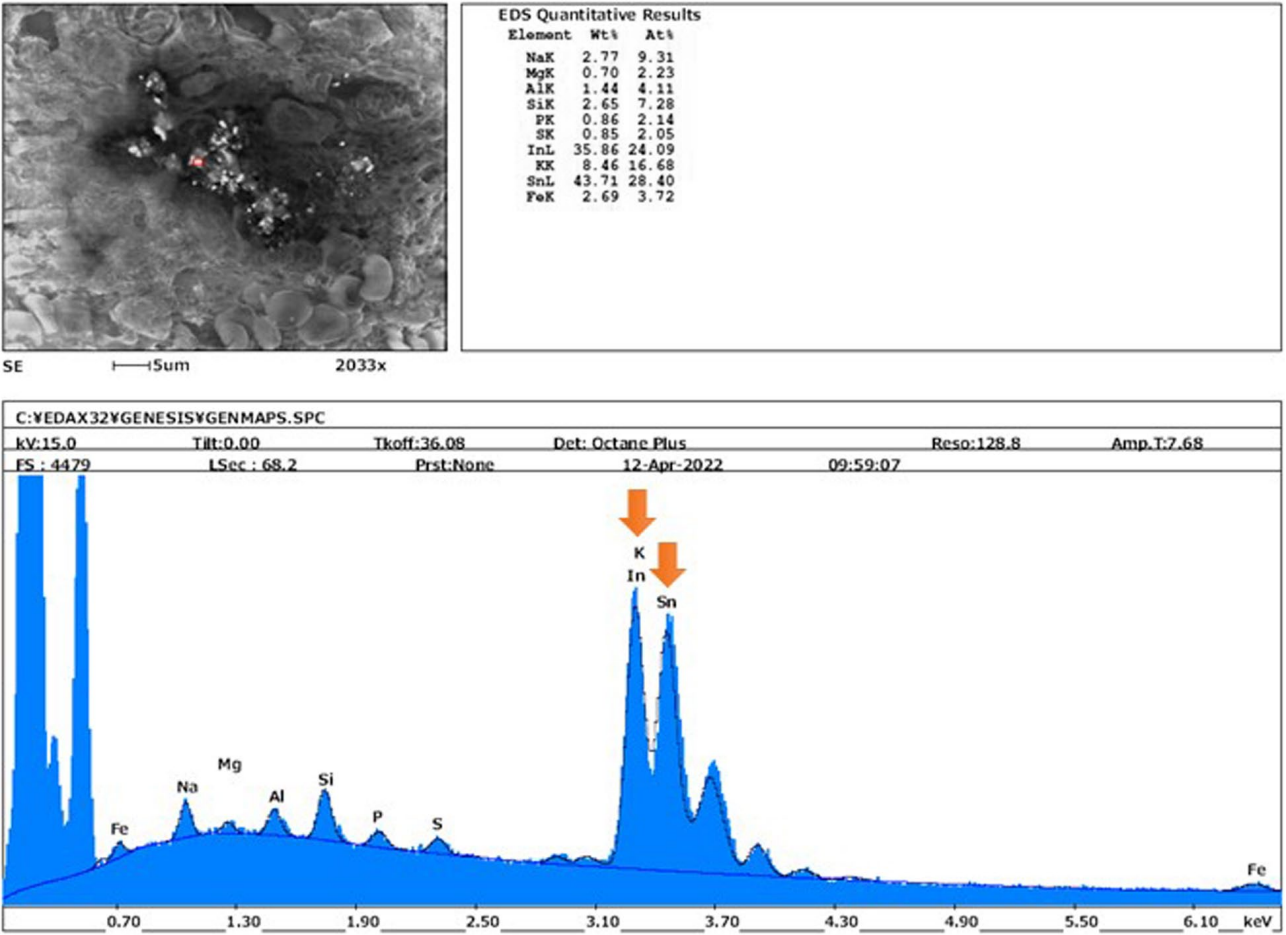
The result of elemental analysis The result of analysis using scanning electron microscopy (SEM) combined with energy dispersive spectroscopy (EDS). The deposited particles contained indium (In) and tin (Sn) (orange arrows)

Analysis using scanning electron microscopy with an energy dispersive X-ray spectroscopy (SEM–EDX, SU3500, Hitachi High-Technologies GLOBAL, Japan) at the Center of Advanced Instrumental Analysis, Kyushu-University revealed that the deposited particles contained indium and tin (Fig. 5). This result supported ITO deposition in the lung and was compatible with the history of ITO exposure and the deposition remains for decades.

## Discussion and conclusion

In our case, general histological findings of indium lung, such as emphysema and fibrosis with deposition of particles and cholesterol granulomas, were observed throughout the patient’s lung. Particularly, a characteristic histological feature of our case was a mixture of severe distal acinar emphysema and severe perilobular fibrosis showing a honeycomb pattern. Nakano et al. reported the indium lung patient who had a smoking history and underwent bilateral LTX eight years after the nine-year ITO exposure ended [6, 7]. That patient’s lung also showed severe emphysema and interstitial fibrosis including fibroblastic foci [6]. They described pan-lobular cystic changes in the emphysematous area but did not mention the subtype of emphysema and a histological pattern of the fibrosis in the report. Therefore, severe emphysema and fibrosis might be common histological features of indium lung, especially with long-term history, but to elucidate more precise histological features of long-term indium lung, including the subtype of emphysema and the pattern of interstitial fibrosis, further accumulation of cases is required. “Emphysema with fibrosis” is often associated with airspace enlargement with fibrosis (AEF). AEF is a smoking-related condition that shows multiple thin-walled cystic lesions with bronchiocentric locations, interstitial fibrosis with structural remodeling, and centrilobular emphysema, sometimes accompanied by respiratory bronchiolitis [8]. Our case does not match the characteristics of AEF because this patient is a nonsmoker, and histologically, the specimen showed distal acinar emphysema, and the cystic airspace enlargement is observed primarily in subpleural, not attributable to respiratory bronchioles. The exact mechanism of pathogenesis of distal acinar emphysema is still unclear, but the connection between the pleura and the alveoli may be fragile due to the low distribution of capillaries and elastic fibers [9]. Neutrophilic inflammation is reported to be significantly associated with distal acinar emphysema in chronic obstructive pulmonary disease patients [10]. Indium could activate the NLR family pyrin domain containing 3 inflammasome, which might induce persistent neutrophilic inflammation [11, 12]. Hence, indium could cause persistent neutrophilic inflammation, which might damage the connection between the pleura and the alveoli, and result in distal acinar emphysema. And in our case, severe peri-lobular fibrosis showing honeycomb changes, which are one of the most important features of a UIP pattern, were observed with/without peribronchial involvement. Asbestosis, a famous occupational lung disease showing a UIP pattern, was used to be believed that started in the respiratory bronchiole and gradually extended outwards to the peripheral lobules, but now a UIP pattern in asbestosis is known to occur regardless of the degree of peribronchial fibrosis [13]. Further examinations are needed to elucidate the pathogenesis of honeycombing fibrosis and distal acinar emphysema in indium lung.

Cummings et al. reported two cases of indium lung which showed pulmonary alveolar proteinosis (PAP) [14]. In a review of 10 cases of indium lung, diagnoses of interstitial lung disease (ILD) were made 4–13 years after first exposure, and those of PAP were made 1–2 years after first exposure [15]. Only one case of PAP had serum GM-CSF autoantibody. Cholesterol is a well-known component of pulmonary surfactant, but, interestingly, cholesterol clefts were pathologically observed in both the indium lung patients with/without PAP. In the surveillance of ITO workers in Japan, localized PAP-like findings were observed, but elevations of serum GM-CSF autoantibodies were not detected [16]. In animal models, PAP is a common feature caused by exposure to ITO. PAP appeared in the early phase after exposure of rats to indium [11, 12]. PAP and acute inflammation were followed by lung fibrosis and emphysema, and the severity of these lesions worsened in a dose- and time-dependent manner [11]. But autoantibody for GM-CSF was not detected in the indium treatment group [12]. Therefore, PAP might appear in the very early phase of indium lung in a manner independent of GM-CSF antibodies. In our case, we could detect little surfactant in the entrapped airspace, but the surface of cholesterol clefts was positive for SP-A, and it might indicate that cholesterol clefts were derived from surfactants. In the case report of autoimmune PAP with fibrosis, the fibrotic area contains less surfactant than other areas do [17]. Therefore, our case may have once presented PAP before the first VATs.

The previous reports using animal models demonstrated that ITO particles resided and kept injuring the lung tissue for weeks or months, and only a little ITO was transferred to other organs [18, 19]. There are few reports about the long-term influence of ITO on humans. Amata, et al. revealed that high levels of indium exposure were related to progressive emphysematous changes through examinations of 84 workers from 2002 to 2010 [20]. They also reported that the biological half-life of serum indium in a human was estimated to be 8.09 years. And, ITO is now considered to be a risk factor for lung cancer because an ex-ITO worker without smoking history was diagnosed with primary lung cancer 17 years after the exposure stopped [5]. Recently, the surveillance of the cohort with low dose exposure to asbestos revealed that low dose exposure to asbestos was a risk factor for interstitial lung abnormalities. In the cohort with interstitial lung abnormalities, the median of estimated cumulative asbestos exposure was 0.7 fiber/ml‐year, and the mean of time since first asbestos exposure was 53.5 years [20]. In our case, the decline of respiratory function clinically progressed about 20 years after exposure to ITO ended. Pathologically, emphysema and fibrosis significantly progressed and cholesterol granulomas increased in the recipient lung of LTX, compared to VATS specimens. Particles containing In and Sn are still deposited in the recipient lung tissue of our case and the case of the previous report [6]. Therefore, ITO may reside in the lung for a long time and keep injuring the tissue, it may result in a progressive decline of respiratory function. In this case, the patient-derived left lung remains, and it is necessary to monitor for further development of emphysema, interstitial pneumonia, pneumothorax, and lung cancer. And asymptomatic workers with a history of exposure to ITO also need regular medical follow-ups.

We herein firstly reported the histopathology of an indium lung without a smoking history, which progressed 20 years after cessation of exposure. The lung tissue showed severe subpleural destruction of lung tissue such as distal acinar emphysema and honeycomb changes. These histological findings might contribute to the histological evaluation of the severity of indium lung. Because of the progressive nature of indium lung, careful follow-up is recommended for workers exposed to ITO even if they are asymptomatic.

## Data Availability

Not applicable.

## Abbreviations

ITO: Indium-tin oxide
LTX: Lung transplantation
FEV1: Forced expiratory volume in one second
FVC: Forced vital capacity
DLco: Diffusing capacity for carbon monoxide
VATS: Video-assisted thoracoscopic surgery
KL-6: Krebs von den lungen 6
HRCT: High-resolution computerized tomography
GGO: Ground glass opacity
GM-CSF: Granulocyte–macrophage colony-stimulating factor
SP-A: Surfactant protein-A
UIP: Usual interstitial pneumonia
PAS: Periodic acid Schiff
PAP: Pulmonary alveolar proteinosis
ILD: Interstitial lung disease
AEF: Airspace enlargement with fibrosis
